# The Union Oil Stove

**Published:** 1873-01

**Authors:** 


*The Union Oil Stove.*

The above stove is admirably adapted to the wants of the sick room. It is small, burns kerosene oil, is easily managed, and is, altogether, one of the greatest conveniences of the kind we have ever seen. We have been using one, and like it. For sale by F. H. LeDuc, Atlanta, Georgia.

